# Cost-effectiveness of LiveLighter® - a mass media public education campaign for obesity prevention

**DOI:** 10.1371/journal.pone.0274917

**Published:** 2022-09-21

**Authors:** Jaithri Ananthapavan, Huong Ngoc Quynh Tran, Belinda Morley, Ellen Hart, Kelly Kennington, James Stevens-Cutler, Steven J. Bowe, Paul Crosland, Marj Moodie

**Affiliations:** 1 Deakin Health Economics, School of Health and Social Development, Institute for Health Transformation, Deakin University, Geelong, Victoria, Australia; 2 Global Centre for Preventive Health and Nutrition, School of Health and Social Development, Institute for Health Transformation, Deakin University, Geelong, Victoria, Australia; 3 Centre for Behavioural Research in Cancer, Cancer Council Victoria, Melbourne, Victoria, Australia; 4 Cancer Council Western Australia, Subiaco, Western Australia, Australia; 5 Deakin Biostatistics Unit, Faculty of Health, Deakin University, Geelong, Victoria, Australia; US Department of Agriculture, UNITED STATES

## Abstract

**Background:**

The Western Australian LiveLighter® program has implemented a series of mass media advertising campaigns that aim to encourage adults to achieve and maintain a healthy weight through healthy behaviours. This study aimed to assess the cost-effectiveness of the LiveLighter® campaign in preventing obesity-related ill health in the Western Australian population from the health sector perspective.

**Methods:**

Campaign effectiveness (delivered over 12 months) was estimated from a meta-analysis of two cohort studies that surveyed a representative sample of the Western Australian population aged 25–49 years on discretionary food consumption one month pre- and one month post-campaign. Campaign costs were derived from campaign invoices and interviews with campaign staff. Long-term health (measured in health-adjusted life years (HALYs)) and healthcare cost-savings resulting from reduced obesity-related diseases were modelled over the lifetime of the population using a validated multi-state lifetable Markov model (ACE-Obesity Policy model). All cost and health outcomes were discounted at 7% and presented in 2017 values. Uncertainty analyses were undertaken using Monte-Carlo simulations.

**Results:**

The 12-month intervention was estimated to cost approximately A$2.46 million (M) (95% uncertainty interval (UI): 2.26M; 2.67M). The meta-analysis indicated post-campaign weekly reduction in sugary drinks consumption of 0.78 serves (95% UI: 0.57; 1.0) and sweet food of 0.28 serves (95% UI: 0.07; 0.48), which was modelled to result in average weight reduction of 0.58 kilograms (95%UI: 0.31; 0.92), 204 HALYs gained (95%UI: 103; 334), and healthcare cost-savings of A$3.17M (95%UI: A$1.66M; A$5.03M). The mean incremental cost-effectiveness ratio showed that LiveLighter® was dominant (cost-saving and health promoting; 95%UI: dominant; A$7 703 per HALY gained). The intervention remained cost-effective in all sensitivity analyses conducted.

**Conclusion:**

The LiveLighter® campaign is likely to represent very good value-for-money as an obesity prevention intervention in Western Australia and should be included as part of an evidence-based obesity prevention strategy.

## Introduction

Globally, overweight and obesity continue to be an urgent public health problem, to which 4.7 million deaths and almost 150 million disability adjusted life years in 2017 were attributable [1]. The burden in Australia mirrors other high income countries, with 67% of the adult population and 25% of children and adolescents being affected by overweight and obesity [2]. The prevalence of overweight and obesity is even higher in the Western Australian (WA) population, estimated to impact 72% of those over aged 16 years and over in 2019 [3]. In addition to the health burden, there are also significant costs borne by all members of society, estimated to be A$11.8 billion in Australia in 2017–18 [4].

It is well recognised that curbing the high prevalence of overweight and obesity requires multiple actions from all levels of government across multiple sectors, industry, the community and individuals [5, 6]. The World Health Organization and various public health, academic and medical groups in Australia recommend that a crucial action that can be taken by governments is the funding of obesity prevention mass media campaigns to motivate and support healthy behaviours [7, 8]. Mass media campaigns use varied communication channels such as television, radio, online, social media and printed resources to promote public health messages at the population level [9]. The impact of mass media campaigns has been described using a hierarchical change model with proximal indicators related to awareness of the campaign; intermediate indicators related to increased knowledge and understanding of the key messages, increased salience of the key messages, change in attitudes and beliefs about the key messages, increased confidence in the ability to adopt new behaviours, and increased intention to change behaviour; and distal indicators related to short term and long term behaviour change [10]. The effectiveness of mass media campaigns can therefore be measured at various levels with distal indicators providing more robust evidence of intervention effectiveness. A systematic review of mass media campaigns targeting obesity prevention found that campaigns can have an impact on intermediate outcomes such as knowledge and attitudes, however there was limited evidence on distal measures [11].

There have been several mass media campaigns funded by Australian federal and state governments aimed at addressing overweight and obesity-related health behaviours. These campaigns have demonstrated positive impacts on the public’s awareness of key messages and intentions for behaviour change [12–15]. The LiveLighter® mass media campaign has been funded by the Western Australian Department of Health since 2012 and employs multiple strategies and platforms to deliver key messages related to healthy eating, an active lifestyle and the achievement and maintenance of a healthy weight [13, 16]. From 2012–2019, seven television-led phases of the LiveLighter® campaign, each targeting different aspects of unhealthy behaviour and the associated health impact, have aired over 22 campaign periods (referred to as ‘waves’) in WA. The LiveLighter® mass media campaign has also been licensed for use in other Australian states. Studies that evaluated the effectiveness of the Sugary Drinks phase of the LiveLighter® campaign in the states of Victoria and WA showed significant reductions in the frequency of sugary drinks consumption after the campaign [17, 18].

Given that most obesity prevention mass media campaigns are largely publicly funded, the need for robust evaluation including economic appraisal has been identified [11, 19]. However, despite value for money being highlighted in best practice guidance on mass media campaign implementation and evaluation, there are no economic evaluations of Australian mass media programs targeting obesity prevention published in the academic literature [11]. This study aimed to evaluate the effectiveness and the cost-effectiveness of the LiveLighter® campaign aired for one year in WA from the health sector perspective.

## Methods

### The effectiveness of the Western Australian LiveLighter® mass media campaign

The evaluation of real-world interventions is challenging. Many mass media campaigns are evaluated using cross-sectional surveys, however the use of cohort studies has been highlighted as a more robust method for the evaluation of these public health interventions [11]. The effectiveness of LiveLighter® was based on two television-led phases evaluated using a cohort study design (Sugary Drinks (2013) [18] and Junk Food (2016)). These campaign evaluations surveyed a representative sample of the WA population with respect to gender and rurality, aged between 25 and 49 years. Random digit dialling to private household telephone landline numbers was undertaken within one month pre- and post-campaign broadcasting. The survey questions related to proximal and distal indicators of campaign effectiveness including self-reported consumption of various food items over the last seven days, recent changes in consumption and intention to change diet related behaviours. Details of the Sugary Drinks 2013 campaign and the Junk Food 2016 campaign are shown in Table 1. Ethics approval for the collection of this data was obtained from Cancer Council Victoria’s Human Research Ethics Committee (HREC 0018) and verbal informed consent was obtained from participants prior to commencing the telephone survey.

**Table 1:** Details of the LiveLighter® campaigns used to assess effectiveness for the purposes of the economic evaluation.

Campaign	Campaign period (duration in weeks)	Cohort study survey period (sample size)
**Sugary Drinks 2013 [20]**	July-August 2013	May-June 2013 T1 (n = 1 504)
		August-September 2013 T2 (n = 822)
	September-November 2013	
	February-March 2014	
	April–May 2014	
**Junk Food 2016**	April-May 2016	February-April T1 (n = 1 501)
		May-June T2 (n = 737)
	June-July 2016	
	August-October 2016	
	February-April 2017	
	July-October 2017	

S1 Appendix shows the survey questions used to evaluate campaign effectiveness. The reported number of serves of discretionary foods (sugary drinks, fast food and sweet food) consumed by survey respondents were calculated by multiplying the number of days the food was consumed over the week (previous seven days) by the number of times it was consumed each day. Paired t-tests were used to assess the differences in consumption pre- and post-campaign. The results of the two campaigns were combined using fixed effects meta-analysis (see S2 Appendix for more details). All analyses were undertaken using STATA 16.0 [20].

### Estimating the change in energy consumption, weight and body mass index (BMI)

Statistically significant changes in the consumption of discretionary foods from the meta-analysis were used to estimate the change in energy consumption (kilojoules (kJ) consumed per week) and subsequent changes in body weight resulting from the LiveLighter® campaign (see S3 Appendix for details of assumptions and calculations). The change in kJ consumed was assumed to be maintained for the duration that the campaign was aired over the one-year intervention period (three waves, each aired for an average of 7.5 weeks, total duration of 22.5 weeks). The change in kJ consumed was converted to a change in weight using published energy balance equations [21, 22]. Changes in weight were assumed to be maintained for one year (the intervention period). After the one year intervention, it was assumed that weight status reverted back to pre-intervention values. The change in BMI was calculated using age- and gender-specific height profiles for the 2017 WA population [23].

### Estimating the cost of the LiveLighter® campaign

Archived invoices and costing estimates provided by media agencies for all seven television-led LiveLighter® campaigns were used to estimate the average annual costs of the three main components of the campaign: (i) pre-campaign, (ii) production, and (iii) campaign broadcast. Staff hourly rates and time fractions allocated to perform LiveLighter® tasks specific to each position were ascertained from interviews with staff involved in the development and delivery of the most recent campaign phase in WA. Staff on-costs of 14.5% and leave loading of 17.5% were incorporated into the analyses [24]. All costs were reported in A$ 2017 values and adjusted using the Gross Domestic Product Implicit Price Deflator where required [25].

Pre-campaign costs involved staff time to plan and develop the campaign phase idea and testing the concept with members of the general public. Production costs involved development of the advertisement for a new campaign phase and the refreshing of broadcast materials to re-run the campaign over additional waves over the one-year intervention period. The key cost items for the broadcast component included media placement across various channels. The cost for each media channel was estimated from 22 waves of the LiveLighter® campaign. Website hosting and support costs were a fixed annual cost that was included in the production component. The LiveLighter® campaigns have aired between two and three waves over a 12-month period with the majority of phases aired over three waves. It was assumed that the intervention consisted of airing one new campaign with two additional waves of the same campaign re-run over the 12-month period. Table 2 provides an aggregated estimate of the three campaign components and the number of campaign phases that informed the cost estimate. Detailed unit costs have not been provided as the media contracts are confidential.

**Table 2:** Aggregated costs for the different components of the LiveLighter® campaign.

	Cost items	Number of campaign phases used to calculate cost item	Total costs (A$2017)	Distribution
**Pre-campaign**	Concept testing	2 phases	$44 808	Gamma*
	Concept development and animatic creation	2 phases		
**Production** ^**β**^	Pre-campaign/Production staff time costs	1 phase	$688 879	Pert^α^ and Gamma*
	Advertisement production (television, audio including radio and Spotify, cinema, digital—including social media, online, outdoor, press including magazine)	4 phases		
	Creative agency fees	1 phase		
	Web hosting and support	1 phase		
**Campaign broadcast** ^**β**^	Advertisement broadcast (television, radio, cinema, online and search engine marketing, outdoor, press, Indigenous media)	7 phases	$621 427	Pert^α^ and Gamma*
	Media buying and planning fees	7 phases		
	Spot monitoring fees	4 phases		
	Campaign broadcast supporting staff time costs	1 phase		
	Electronic support materials (web content and online tools and resources)	1 phase		

Notes: A$2017: Australian dollars in 2017 values

* A Gamma distribution is often used to model the distribution of variables that have a one sided limit. The Gamma distribution ranges from 0 to infinity and is defined by the alpha and beta that are calculated using the mean and standard deviation.

^α^ A Pert distribution is a re-scaled and re-parametrised Beta distribution and takes the minimum, mode and maximum values to define the distribution.

^β^ Costs are for one wave of a new campaign phase.

Whilst evaluation of mass media campaigns is important to demonstrate their ongoing effectiveness, it was assumed that the intervention was operating in steady state at its full effectiveness potential; therefore, campaign evaluation costs were excluded from the economic evaluation.

### Estimating long-term health and economic outcomes

A widely used and validated proportional, multi-state lifetable Markov cohort model (ACE-Obesity Policy model) was used to estimate the impact of changes in BMI on the epidemiology of obesity- related diseases and long-term health outcomes quantified in health-adjusted life years (HALYs) gained (a summary measure of population health that captures morbidity and mortality impacts [26]). Details of the model have previously been published [5, 27–34] and are described here briefly. Changes in the incidence of nine obesity-related diseases (ischaemic heart disease, hypertensive heart disease, ischaemic stroke, diabetes, and hip and knee osteoarthritis, kidney cancer, colorectal cancer, endometrial cancer, and breast cancer) were calculated using potential impact fractions using relative risks from the Global Burden of Disease study [35]. Disease specific lifetables estimated the morbidity and mortality impacts, and disability weights from the Global Burden of Disease study were used to value the time spent in each of the disease states [36]. The model was modified to simulate the 2017 WA population. Health care costs associated with each of the diseases were ascertained from the Australian Institute of Health and Welfare (AIHW) Disease Costs and Impact Study 2001 [37] inflated to A$2017 using the AIHW Health Price Index [25].

### Estimating the cost-effectiveness of the LiveLighter® program

The incremental cost and benefits of the LiveLighter® intervention were calculated using a ‘no intervention’ comparator from a health sector perspective. The time horizon for the intervention was one year, and the impacts were modelled over the lifetime of the WA population aged 25–49 years. The discount rate recommended for economic evaluations vary across Australian government jurisdictions and across agencies. The most commonly recommended base case discount rate is 7% (with 3% and 10% tested in sensitivity analyses) [38, 39], however various preventive health economic evaluations in Australia have used a lower discount rate of 3% [5], whereas the Pharmaceutical Benefits Advisory Committee (PBAC) use 5% (with 3.5% and 0% used in sensitivity analyses) [40]. Given the decision context of state government resource allocation, a 7% discount rate was used in the base case analysis and all values are reported in 2017 values.

Incremental cost-effectiveness ratios (ICER) were calculated to assess whether the intervention was cost-effective. However, in Australia, the willingness to pay for HALYs is not explicit. A study of prior decisions made by the PBAC found that the likely threshold was between A$65 000 and A$117 000 per Quality Adjusted Life Year (QALY) gained (A$42 000 and A$76 000 in 1998/1999 values [41] inflated to 2017 values using the total health price index deflator [25]). A more recent study found that the majority of drugs with an ICER over A$74 000 (A$52 400 in 2003 values [42] inflated to 2017 values) were not approved. We assumed the value of a QALY is the same as a HALY (the difference between the two measures is the method used to assign utility weights to health states) and took a conservative approach and judged the intervention to be cost-effective if the ICER was below A$65 000 per HALY gained.

### Uncertainty and scenario analyses

Extensive parameter uncertainty analyses were undertaken using Monte Carlo simulations using Ersatz version 1.35 [43], an Excel add-in software. Two thousand iterations of the model were run drawing input values from defined probability distributions (see Tables 2 and 4 for the distribution of costs and effectiveness parameters). Input values and distributions for the ACE-Obesity Policy model have been previously published [5, 27]. All results are presented on a cost-effectiveness plane and tabulated with 95% uncertainty intervals (UIs).

Univariate and multivariate scenario analyses were undertaken to assess the impact of key assumptions related to the population impacted by the intervention, the duration the campaign aired over the one-year intervention, the time horizon for the modelled impacts and the discount rate. Details of the scenario analyses are shown in Table 3. See S4 Appendix for the completed CHEERS checklist.

**Table 3:** Inputs for the base case and scenario analyses.

	Base case	Scenario 1: Population 18+	Scenario 2: campaign aired over 2 waves	Scenario 3: 10-year time horizon	Scenario 4: 3% discount rate
**Modelled WA population**	25–49 years	18–100 years*	25–49 years		
**Campaign duration**	1 year				
**Pre-campaign costs**	A$44 808				
**Production costs**	A$688 879				
**Broadcast costs**	A$1.7M		A$1.2M	A$1.7M	
**Number of waves**	3 waves		2 waves	3 waves	
**Duration of each wave (weeks)**	7.5 weeks^α^				
**Duration of reduced consumption in a year (weeks)**	23.1		15.4	23.1	
**Effect on sugary drink and sweet food consumption**	Meta-analysis of combined effect of Sugary drinks 2013 and Junk Food 2016 campaigns				
**Maintenance of effect**	1 year				
**Model time horizon**	Lifetime			10 years	Lifetime
**Discount rate**	7%				3%

Notes: *Effectiveness was adjusted to reflect the baseline consumption differences between other age groups and the surveyed population (See S5 Appendix);

^α^Modelled using a Pert distribution (minimum 3; maximum 13); A$: Australian dollar in 2017 values M: million

## Results

### The effectiveness of the Western Australian LiveLighter® mass media campaign

Changes in the number of serves of discretionary foods consumed each week post campaign compared to pre campaign are shown in Table 4. The Sugary Drinks 2013 campaign resulted in significant reductions in the consumption of sugary drinks, whilst the Junk Food 2016 campaign resulted in significant reductions in the consumption of both sugary drinks and sweet foods. The meta-analysis found significant reductions in the consumption of sugary drinks (0.78 serves per week, 95% UI: 0.57 to 1.0) and sweet foods (0.28 serves per week, 95% UI: 0.07 to 0.48) (see S2 Appendix for the meta-analysis forest plots).

**Table 4:** Change in consumption of serves per week of discretionary food as a result of the LiveLighter® campaigns.

Food categories	Sample size	Change in number of serves/week post campaign compared to pre campaign (95% UI)	Distribution used in the cost-effectiveness analysis
**Sugary Drinks 2013 LiveLighter® campaign**			
Sugary drinks	821	-0.60 (-0.91 to -0.30)	Not applicable
Fast food	813	0.06 (-0.04 to 0.16)	
Sweet food	630	-0.25 (-0.60 to 0.10)	
**Junk Food 2016 LiveLighter® campaign**			
Sugary drinks	736	-0.96 (-1.26 to -0.66)	Not applicable
Fast food	735	-0.10 (-0.22 to 0.02)	
Sweet food	736	-0.29 (-0.54 to -0.03)	
Salty food	736	-0.13 (-0.29 to 0.04)	
**Meta-analysis results**			
Sugary drinks		-0.78 (-1.00 to -0.57)	Lognormal*
Sweet foods		-0.28 (-0.48 to -0.07)	Lognormal*

Notes: * The lognormal distribution is defined by the mean and the standard error (not reported here); UI: uncertainty interval

The change in consumption of sugary drinks and sweet foods was estimated to result in a mean weight reduction of 0.58kg (95% UI: 0.31 to 0.92). Table 5 shows the modelled weight change for all scenarios.

**Table 5:** Effectiveness, costs and cost-effectiveness results.

	Mean population weight reduction, kg (95% UI)	Pre-campaign and production component costs, A$M (95% UI)	Campaign broadcast component costs, A$M (95% UI)	Total intervention costs, A$M (95% UI)	Total healthcare cost, A$M (95% UI)*	Total net costs, A$M (95% UI)*	Total HALYs gained	ICERs, mean, A$/HALY gained (95% UI)
							(95% UI)	
**Base case**	0.58	0.73	1.73	2.46	-3.17	-0.71	204.15	Dominant
	(0.31 to 0.92)	(0.62; 0.87)	(1.57; 1.90)	(2.26; 2.67)	(-5.03; -1.66)	(-2.55; 0.82)	(103.54; 334.33)	(Dominant; 7 703)
**Scenario 1: Population 18+**	0.59	0.73	1.73	2.46	-6.53	-4.06	470.02	Dominant
	(0.32 to 0.95)	(0.62; 0.87)	(1.57; 1.90)	(2.27; 2.67)	(-10.51; -3.38)	(-8.07; -0.91)	(239.02; 763.89)	(Dominant; Dominant)
**Scenario 2: campaign aired over 2 waves**	0.39	0.73	1.18	1.91	-2.13	-0.21	137.02	Dominant
	(0.20 to 0.62)	(0.62; 0.87)	(1.07; 1.28)	(1.76; 2.08)	(-3.46; -1.11)	(-1.56; 0.84)	(70.66; 227.45)	(Dominant; 11 869)
**Scenario 3: 10-year time horizon**	0.58	0.73	1.73	2.46	-1.33	1.13	84.83	13 362
	(0.31 to 0.93)	(0.62; 0.87)	(1.57; 1.90)	(2.27; 2.67)	(-2.11; -0.71)	(0.33; 1.79)	(43.33; 140.33)	(2 441; 40 671)
**Scenario 4: 3% discount rate**	0.58	0.73	1.73	2.46	-5.95	-3.5	405.67	Dominant
	(0.31 to 0.92)	(0.62; 0.87)	(1.57; 1.90)	(2.26; 2.67)	(-9.69; -3.07)	(-7.23; -0.60)	(207.85; 667.18)	(Dominant; Dominant)

Notes: A$: Australian dollar in 2017 values; HALY: health-adjusted life year; ICER: incremental cost-effectiveness ratio; LY: life year; UI: Uncertainty interval

* Negative costs represent cost-savings

### Cost and cost-effectiveness of the LiveLighter® campaign

The LiveLighter® campaign was estimated to cost A$2.46 million (M) (95% UI: A$2.26M to A$2.67M) over one year, with approximately 70% of the costs accrued during the campaign broadcast component. The cost of the campaign reduced to A$1.91M (95% UI: A$1.76M to A$2.08M) when it was assumed that the campaign aired for two waves over the year rather than three (Scenario 2, Table 5).

**Fig 1:**
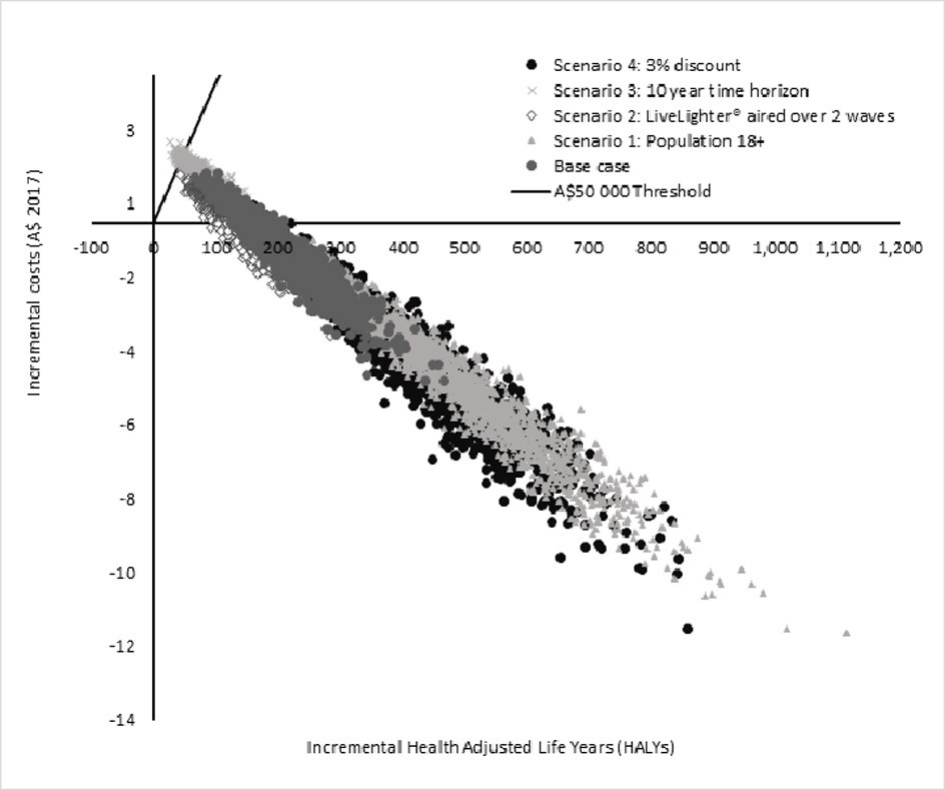
Cost-effectiveness plane of base case and sensitivity analyses.

The LiveLighter® campaign was estimated to result in 204 HALYs gained (95%UI: 104 to 334) and healthcare cost-savings of A$3.17M (95%UI: A$1.67M to A$5.03M) over the lifetime of the WA population aged 25–49 years. Net health gains and cost-savings meant that the intervention was dominant (95% UI: dominant to A$7 703) in 76% of model iterations and cost-effective in 100% of model iterations (see Table 5 and Fig 1).

The ICER was dominant in all scenarios evaluated except in Scenario 3 when the model time horizon was limited to 10 years (ICER: A$13 362, 95%UI: A$2 441 to A$40 671). In Scenario 2, the less intense campaign (i.e. two waves of the campaign in a year rather than three) was approximately 22% less costly to implement, however this resulted in a 33% reduction in health benefits. This resulted in less favourable cost-effectiveness results compared to the base case, however the intervention is still considered cost-effective in accordance with the threshold. When a lower discount rate was used (Scenario 4), the HALYs gained and healthcare cost-savings almost doubled (increased by a factor of 1.99 and 1.88 respectively) (Table 5).

## Discussion

This analysis found that the LiveLighter® campaign results in reductions in self-reported consumption of sugary drinks and sweet foods following campaign airings. This is estimated to result in reduced weight and long-term health benefits and cost-savings from avoiding obesity-related diseases. Various plausible scenarios were tested to assess the impact on the cost-effectiveness results. All scenarios tested showed that the LiveLighter® campaign is either a highly cost-effective or dominant intervention (cost-saving and health promoting). Broadening the population impact of the intervention (Scenario 1) to include all WA adults resulted in additional health and healthcare cost-savings, however there was large variability in the results demonstrated by the wider spread of the ICER results as can be seen on the cost-effectiveness plane in Fig 1. Since 2012, there have been two occasions when the LiveLighter® campaign aired over two waves in a year rather than three. Scenario 2 showed that this is likely to result in a less favourable cost-effectiveness results with the reduction in costs outweighed by the reduced benefits, however the intervention remained dominant. Given that there is reduced certainty of events many years into the future, Governments are interested in the shorter term impacts on interventions [44]. The short-term impacts of the intervention over 10 years (Scenario 3), showed that the intervention remained cost-effective with an ICER well below the threshold value used for Australia. The impact of the discount rate on preventive health interventions is highlighted in Scenario 4 where the lower rate was predicted to almost double the health gains and healthcare cost-savings.

To our knowledge this is the first economic evaluation of a mass media campaign promoting healthy weight published in the academic literature. A recent paper reporting the overall results of the Australian priority-setting study using the ACE-Obesity Policy model [5] predicts that a modelled mass media campaign related to sugary drinks consumption implemented at the national level would be dominant, however full details of the evaluation are not yet published. A recent systematic review of economic evaluations of public health interventions related to physical activity and diet included two studies reporting on the cost-effectiveness of mass media campaigns related to physical activity and salt reduction–in both studies, mass media campaigns were found to be dominant [45]. The lack of reporting of the economic credentials of obesity prevention mass media campaigns has been highlighted as a limitation of previous campaigns, and therefore this study provides important insights for policymakers [11].

A key strength of this analysis is the use of a widely used and validated economic model to estimate the economic credentials of the LiveLighter® program in WA [5]. This study highlighted that prevention interventions that effectively change behaviour have the potential to be highly cost-effective, resulting in reductions in obesity-related diseases and healthcare cost-savings. Given that the current obesity epidemic requires a suite of obesity prevention interventions, this study demonstrates that mass media programs should be included in a package of evidence-based, cost-effective interventions.

The key limitation of this study relates to the precision of the measure to assess the effectiveness of the LiveLighter® campaign. Firstly, despite seven phases of the LiveLighter® campaign in WA since 2012, only two phases have been evaluated using a cohort design. There were also limitations in the cohort study design, with the absence of a control group that was not exposed to the LiveLighter® campaign. Case-control study designs provide stronger evidence of intervention effect, however they are less commonly used to evaluate mass media campaigns, likely due to the increased costs associated with their implementation [11]. The need for these more powerful study designs should be communicated to policymakers and adequate funding for well-designed and conducted evaluations should be factored into funding for mass media campaigns. When future campaign evaluation results are available, an updated meta-analysis to estimate the overall effectiveness of LiveLighter® campaign should be undertaken. Secondly, self-reported behaviour change is subject to recall and self-report bias and the behaviour change questions in the survey meant additional assumptions related to serve size were required. Future surveys should also aim to estimate the quantity of foods consumed rather than frequency of consumption only. It was assumed that the changes in consumption were maintained for the duration the campaign aired (approximately 23 weeks over the one-year intervention). This may be an underestimate as mass media campaigns can help establish new social norms and therefore may have long lasting impacts on behaviour change [46]. This should be tested by collecting longer term follow up data to ascertain the effectiveness of ongoing mass media campaigns.

The policy implications for this research are that mass media campaigns such as LiveLighter® are effective in changing dietary behaviour at least in the short term, and are likely to be highly cost-effective. Our analysis showed that campaigns that are sustained for longer are likely to be more cost-effective. This is supported by a recent review that found mass media campaigns that are aired for longer durations at higher intensity are more likely to be effective [9].

## Conclusion

This study demonstrated the economic credentials of public health mass media campaigns targeting diet related health behaviours. The LiveLighter® mass media campaign in WA was found to be both health promoting and cost-saving and therefore represents excellent value for money. Mass media campaigns should be included as part of an evidence-based obesity prevention strategy.

## Supporting information

S1 Appendix Survey questions for the LiveLighter® campaign. (DOCX)

S2 Appendix Meta-analysis methods and forest plots. (DOCX)

S3 Appendix Assumptions used to estimate change in kilojoule consumption resulting from the LiveLighter® campaign. (DOCX)

S4 Appendix The Consolidated Health Economic Evaluation Reporting Standards (CHEERS) checklist. (DOCX)

S5 Appendix Effectiveness of the LiveLighter® campaign on the Western Australian population aged 18 years and over. (DOCX)
