# Supplementary material for: Cost-effectiveness of LiveLighter® - a mass media public education campaign for obesity prevention
Source: PLoS One. 2022 Sep 21;17(9):e0274917. doi: 10.1371/journal.pone.0274917 (PMC9491524; doi:10.1371/journal.pone.0274917)
Supplement: S1 Appendix — (DOCX) [file pone.0274917.s001.docx]

**S1 Table: Survey questions for the LiveLighter® campaign**

| **Item** | **Question** | **Answer option** |
| --- | --- | --- |
| **D1** | Thinking back over the past 7 days, how many serves of fruit did you usually eat each day? A serve of fruit is equal to one medium piece, two small pieces of fruit or one cup of diced fruit.  (Single response). | Serves per day _______  0. None  97. Less than one a day  98. (Don’t know/Can’t say) (Don’t read out)  99. (Refused) (Don’t read out) |
| **D2** | Thinking back over the past 7 days, how many serves of vegetables did you usually eat each day? A serve of vegetables is equal to half a cup of cooked vegetables or 1 cup of salad.  (Single response). | Serves per day _______  0. None  97. Less than once a day  98. (Don’t know/Can’t say) (Don’t read out)  99. (Refused) (Don’t read out) |
| **D3a** | During the past 7 days, on how many days did you drink a can, bottle or glass of a sugar-sweetened beverage such as soft drinks, energy drinks, fruit drink, sports drinks and cordial)?  [INTERVIEWER NOTE: Fruit drink does not include 100% fruit juice.] | Days in the past 7 drank soft drink __________ (0 Skip to D3c)  98. (Don’t know/Can’t say) (Don’t read out) (Skip to D3c)  99. (Refused) (Don’t read out) (Skip to D3c) |
| **D3b**  ***(IF D3a = 1-7)** | On days that you did drink sugar-sweetened beverages, how many times per day did you usually drink them?  (Read out, single response). | 1. Once a day  2. Twice a day  3. 3 times per day  4. 4 or more time per day  98. (Don’t know/Can’t say) (Don’t read out)  99. (Refused) (Don’t read out) |
| **D3c** | In the past month, did you drink more or less sugar-sweetened beverages than usual?  PROMPT: Was that a lot less/more or a little less/more? | 1. A lot less  2. A little less  3. About the same  4. A little more  5. A lot more  98. (Don’t know/Can’t say) (Don’t read out)  99. (Refused) (Don’t read out) |
| **D4a** | During the past 7 days, on how many days did you drink a can, bottle or glass of a diet drink such as diet soft drinks, diet energy drinks or diet cordial)? | Days in the past 7 drank diet soft drink __________ (0 Skip to D4c)  98. (Don’t know/Can’t say) (Don’t read out) (Skip to D4c)  99. (Refused) (Don’t read out) (Skip to D4c) |
| **D4b**  ***(IF D4a = 1-7)** | On the days that you did drink diet drinks, how many times per day did you usually drink them?  (Read out, single response). | 1. Once a day  2. Twice a day  3. 3 times per day  4. 4 or more time per day  98. (Don’t know/Can’t say) (Don’t read out)  99. (Refused) (Don’t read out) |
| **D4c** | In the past month, did you drink more or less diet drinks than usual?  PROMPT: Was that a lot less/more or a little less/more? | 1. A lot less  2. A little less  3. About the same  4. A little more  5. A lot more  98. (Don’t know/Can’t say) (Don’t read out)  99. (Refused) (Don’t read out) |
| **D4d** | How much water do you usually drink each day? This can be plain tap water, mineral water or bottled water. (1 cup = 250ml, 1 average bottle = 2 cups, 1 litre bottle = 4 cups). | 1. I don’t drink water  2. Less than one cup a day  3. About 1 cup a day  4. About 2 cups a day  5.About 3 cups a day  6. About 4 cups a day  7. About 5 cups or more a day  98. (Don’t know/Can’t say) (Don’t read out)  99. (Refused) (Don’t read out) |
| **D4e** | In the past month, did you drink more or less water than usual?  PROMPT: Was that a lot less/more or a little less/more? | 1. A lot less  2. A little less  3. About the same  4. A little more  5. A lot more  98. (Don’t know/Can’t say) (Don’t read out)  99. (Refused) (Don’t read out) |
| **D5a** | During the past 7 days, on how many days did you eat take-away or ‘fast foods’ (such as fish and chips, hamburgers, fried chicken, pizza, sausage rolls, meat pies)? | Days in the past 7 ate takeaway _______________(0 days Skip to D6a)  98. (Don’t know/Can’t say) (Don’t read out) (Skip to D6a)  99. (Refused) (Don’t read out) (Skip to D6a) |
| **D5b**  ***(IF D5a = 1-7)** | On days that you did eat take-away or ‘fast food’, how many times per day did you usually eat it?  (Read out) | 1. Once a day  2. Twice a day  3. 3 times per day  4. 4 or more times per day  98. (Don’t know/Can’t say) (Don’t read out)  99. (Refused) (Don’t read out) |
| **D6a** | During the past 7 days, on how many days did you eat sweet foods (such as cakes, biscuits, lollies and chocolates)? | Days in the past 7 ate sweet foods __________ (0 Skip to D10)  98. (Don’t know/Can’t say) (Don’t read out) (Skip to D10)  99. (Refused) (Don’t read out) (Skip to D10) |
| **D6b**  ***(IF D6a=1-7)** | On days that you did eat sweet foods, how many times per day did you usually eat it?  (Read out) | 1. Once a day  2. Twice a day  3. 3 times per day  4. 4 or more times per day  98. (Don’t know/Can’t say) (Don’t read out)  99. (Refused) (Don’t read out) |
| Notes: when not specified, the survey question was applied to all participants; ml: millilitre.  Source: Personal communication with Cancer Council Victoria. | | |

**Sample calculation for number of serves of sugary drinks consumed each week:**

Answer to question D3a * answer to D3b.
