# Supplementary material for: Cost-effectiveness of LiveLighter® - a mass media public education campaign for obesity prevention
Source: PLoS One. 2022 Sep 21;17(9):e0274917. doi: 10.1371/journal.pone.0274917 (PMC9491524; doi:10.1371/journal.pone.0274917)
Supplement: S2 Appendix — (DOCX) [file pone.0274917.s002.docx]

**S2 File: Meta-analysis methods and forest plots**

A cohort study was conducted to assess the impact of the LiveLighter® campaign. Surveys were administered to participants pre and post campaign to assess change in consumption of particular foods. The survey participants were aged between 25 and 49 and representative of the Western Australian population, in terms of gender and residence (metropolitan or rural) (1). Paired t-test results showed the change in consumption of sugary drinks and sweet foods for the Sugary Drinks 2013 and the Junk Food 2016 campaigns. A meta-analysis was conducted to combine the results and to demonstrate the overall impact of LiveLighter® on sugary drink and sweet food consumption. Forest plots were produced to visualise the meta-analysis results. The analyses were conducted using Stata SE version 16 software using the **meta** package (2, 3). The two meta-analyses shown in Figures A and B considered both a random effects (using restricted maximum likelihood method) model and a fixed-effects (inverse-variance weighted) model. Since the test of heterogeneity in both models were not statistically significant at an alpha levels of 5% and 10% we have assumed that the two campaigns shared a common effect size. This means that the differences in the consumption levels between the two campaigns were presumed to be due to sampling error. In both Figure A and B, the overall estimates were the results from fixed effect model and the Random(reml) estimates were results from random effect model. Although, we do note that the I^2^ in the first meta-analysis of change in sugary drink consumption was 62.9% (Figure A) suggests a moderate level of heterogeneity, but since there were only two studies being considered we decided that the overlapping 95% CIs of the two effect sizes from each study was valid justification for a fixed effects model. In Figure A we also note the similar overall effect sizes for the fixed effects and random effects models but with a slightly wider 95% CI for the random effects model. While in Figure B since I^2^ (percentage of the variability in effect estimates that is due to heterogeneity) is zero the overall effect size results from both models are the same. The weight assigned to each study was based on the inverse of the variance of the effect sizes from the two studies. The combined effect sizes were calculated using the weighted mean across the two studies. No sensitivity analysis were undertaken to assess whether any one study skewed the results. The overall effect size results were considered statistically significant when p<0.05.

The results in Figure A and B for the z-test (test of theta θ=0) suggest the overall combined effect sizes were statistically significant and were not equal to zero, since the p-values (in both Figures) were statistically significant (p<0.05) and the 95% Confidence Intervals do not cross zero. Results from the meta-analyses showed that on average, frequency of consumption of sugary drink reduced by 0.78 serves per week (95% CI: 0.57 to 1.00) (Figure A) and sweet food by 0.28 serves per week (95% CI: 0.07 to 0.48) (Figure B).

*Figure A: Meta-analysis of change in sugary drink consumption (serves per week) from the Sugary Drinks 2013 and Junk Food 2016 campaigns*

Notes: Overall: results of the fixed effects model; Random(reml): results of the random effects model; CI: confidence intervals; Diff: difference

*Figure B:* *Meta-analysis of change in sweet food consumption (serves per week) from the Sugary Drinks 2013 and Junk Food 2016 campaigns*

**

Notes: Overall: results of the fixed effects model; Random(reml): results of the random effects model; CI: confidence intervals; Diff: difference

**References**

1. Morley B, Niven P, Dixon H, et al. Association of the LiveLighter mass media campaign with consumption of sugar‐sweetened beverages: Cohort study. *Health Promotion Journal of Australia.* 2019;30(Suppl 1):34.

2. *Stata Statistical Software: Release 16* [computer program]. TX: StataCorp LLC: College Station; 2019.

3. Higgins J, Thompson S, Deeks J, Altman D. Statistical heterogeneity in systematic reviews of clinical trials: a critical appraisal of guidelines and practice. *Journal of health services research & policy.* 2002;7(1):51-61.
