# Supplementary material for: Cost-effectiveness of LiveLighter® - a mass media public education campaign for obesity prevention
Source: PLoS One. 2022 Sep 21;17(9):e0274917. doi: 10.1371/journal.pone.0274917 (PMC9491524; doi:10.1371/journal.pone.0274917)
Supplement: S3 Appendix — (DOCX) [file pone.0274917.s003.docx]

**S3 File: Assumptions used to estimate change in kilojoule consumption resulting from the LiveLighter® campaign**

The following assumptions were used to calculate the change in kilojoule (kJ) consumption based on the change in the number of serves of sugary drinks (such as soft drinks, energy drinks, fruit drink, sports drinks and cordial) and sweet foods (such as cakes, biscuits, lollies and chocolates) consumed over a week.

- Each time sugary drink/sweet food was consumed, the amount consumed was equivalent to one serve size for that particular food item.
- The serve size for sugary drinks was equal to one metric cup (250ml).
- The LiveLighter® survey (see Appendix 1, question D6a) focussed on four main sweet food groups: cakes, biscuits, lollies and chocolate. We used the serve size for each of these foods as recommended by the National Health and Medical Research Council (NHMRC) and the Commonwealth Department of Health [1]. The recommended serve size is based on the serve size for various discretionary foods that provide 600kJs [1]. However, the recommended serve size is often smaller than single serve package sizes for some of these foods. For example, the recommended serving size for chocolate is 25g [1] whereas the size of a regular chocolate bar is over 50g [2]. Therefore the calculated impact of the LiveLighter® on consumption is likely underestimated.
- The Australian Food, Supplement and Nutrient Database (AUSNUT) [3] was used to estimate the energy content of these foods. The kJ per serve of each foods included in the AUSNUT database classified as a sugary drink (cordials, soft drinks, and flavoured mineral waters, electrolytes, energy and fortified drinks), cake, biscuit or lollies was extracted. The mean energy for the specified serve size with uncertainty intervals for all included items were used in the calculation of the change in kJ resulting from the intervention (see Table A).
- The reduction in energy intake was assumed to be maintained for the duration the campaign aired (average duration of one wave was 7.5 weeks, range 5-13 weeks, average of 3 waves over a one year period). The reduction in energy intake was translated into reduction in weight based on published energy balance equations reported by Hall and colleagues [4].
- The weight reduction was assumed to be maintained for one cycle of the model (one year). This is also the duration of the modelled LiveLighter® campaign intervention.
- Given that the cohort study surveys did not show an increase in the consumption of healthy or other foods, it was assumed that there was no compensatory consumption of healthy or other foods resulting from the reduction in consumption of sugary drinks and sweet foods.
- The reduction in BMI was calculated using the height profile of the 2017 Western Australian population, stratified by age group and gender [5].

*Table A: Serve sizes and means kilojoules per serve of sugary drinks and sweet foods*

| **Drink and food groups** | **Serve size** | **Mean kJ per serve (minimum and maximum values used in uncertainty analysis)** | **Distribution used in uncertainty analysis*** | **Food group codes from AUSNUT [3]** | **Number of food items from AUSNUT used to estimate kJ/serve [3]** |
| --- | --- | --- | --- | --- | --- |
| **Sugary drinks** (i.e. cordials, soft drinks, and flavoured mineral waters, electrolytes, energy and fortified drinks) | 250ml (i.e. 1 metric cup) | **982.17**  **(83.59; 2,461.36)** | Pert | 113, 114, 115, 116 | 181 |
| **Sweet foods** |  | **604.22**  **(521.54; 691.06)** | Pert |  |  |
| Cake | 40g (i.e. one slice) | 590.69  (388.73; 797.36) | Pert | 133 | 199 |
| Biscuits | 35g (i.e. 2-3 biscuits) | 660.67  (552.67; 757.38) | Pert | 131 | 84 |
| Lollies | 40g (i.e. 5-6 small lollies) | 650.63  (457.57; 890.12) | Pert | 284 | 31 |
| Chocolate | 25g (i.e. ½ small bar) | 514.89  (425.38; 607.50) | Pert | 281 | 58 |
| Notes: AUSNUT: Australian Food, Supplement and Nutrient Database; g: gram; kJ: kilojoule; ml: millilitre; * A Pert distribution is a re-scaled and re-parametrised Beta distribution and takes the minimum, mode and maximum values to define the distribution. The mean value was used as the mode input in the uncertainty distribution.  Source: Food Standards Australia and New Zealand 2016 [3] | | | | | |
|  | | | | | |

**References**

1. National Health and Medical Research Council, Deparment of Health. Discretionary food and drink choices Canberra2017 [updated 16 May; cited 2020 4 June]. Available from: <https://www.eatforhealth.gov.au/food-essentials/discretionary-food-and-drink-choices#:~:text='Discretionary'%20foods%20and%20drinks%20include,and%2For%20salt%20content%3B%20commercially>.

2. Lal A, Peeters A, Brown V, Nguyen P, Tran HNQ, Nguyen T, et al. The Modelled Population Obesity-Related Health Benefits of Reducing Consumption of Discretionary Foods in Australia. Nutrients. 2020;12(3):649. doi: 10.3390/nu12030649. PubMed PMID: 32121199.

3. Food Standards Australia and New Zealand. AUSNUT 2011-2013 2016 [updated April; cited 2020 4 June]. Available from: <https://www.foodstandards.gov.au/science/monitoringnutrients/ausnut/pages/default.aspx>.

4. Hall KD, Sacks G, Chandramohan D, Chow CC, Wang YC, Gortmaker SL, et al. Quantification of the effect of energy imbalance on bodyweight. The Lancet. 2011;378(9793):826-37.

5. Australian Bureau of Statistics. Microdata: National Health Survey, 2017-18. In: Australian Bureau of Statistics, editor. Canberra: DataLab; 2019.
