# Supplementary material for: Cost-effectiveness of LiveLighter® - a mass media public education campaign for obesity prevention
Source: PLoS One. 2022 Sep 21;17(9):e0274917. doi: 10.1371/journal.pone.0274917 (PMC9491524; doi:10.1371/journal.pone.0274917)
Supplement: S5 Appendix — (DOCX) [file pone.0274917.s005.docx]

**S5 File: Effectiveness of the LiveLighter® campaign on the Western Australian population aged 18 years and over**

Scenario 1 assumed that the LiveLighter® campaign was effective in reducing consumption across all adults living in Western Australia. However, the effectiveness data were from a survey administered to adults aged 25-49 years. We adjusted the reduction in consumption based on the baseline consumption for the different age groups relative to the base case population of 25-49 year olds. (Table B and Table C).

Table B: LiveLighter® campaign impacts on sugary drinks consumption for the Western Australian adult population aged 18 years and over

|  | **Males** | | **Females** | |  |
| --- | --- | --- | --- | --- | --- |
| **Age** | **Reduced consumption (cups per week)** | **Reduced kJ per week** | **Reduced consumption (cups per week)** | **Reduced kJ per week** | **Distribution used in uncertainty analysis*** |
| 18-19 years | 0.91 | 860.26 | 1.55 | 1466.54 | Lognormal |
| 20-24 years | 1.42 | 1349.26 | 1.86 | 1759.94 | Lognormal |
| 25-34 years | 0.82 | 781.46 | 0.66 | 621.12 | Lognormal |
| 35-44 years | 0.92 | 873.21 | 0.97 | 920.11 | Lognormal |
| 45-54 years | 0.60 | 572.50 | 0.72 | 685.93 | Lognormal |
| 55-64 years | 0.44 | 421.85 | 0.24 | 227.46 | Lognormal |
| 65-74 years | 0.51 | 480.81 | 0.93 | 880.84 | Lognormal |
| 75-100 years | 0.33 | 308.34 | 0.66 | 626.26 | Lognormal |
| Average in LiveLighter® survey population | 0.78 |  | 0.78 |  |  |
| Notes: kJ: kilojoule; * Lognormal distribution defined by mean and standard errors from Australian Bureau of Statistics (ABS) mean sugary drinks consumption data.  Source: ABS 2019 [1] | | | | | |

*Table C: LiveLighter® campaign impacts on sweet foods consumption for the Western Australian adult population aged 18 years and over*

|  | **Males** | | **Females** | |  |
| --- | --- | --- | --- | --- | --- |
| **Age** | **Reduced consumption (serves per week)** | **Reduced kJ per week** | **Reduced consumption (serves per week)** | **Reduced kJ per week** | **Distribution used in uncertainty analysis*** |
| 18-19 years | 0.24 | 142.87 | 0.37 | 226.69 | Lognormal |
| 20-24 years | 0.29 | 175.65 | 0.31 | 185.56 | Lognormal |
| 25-34 years | 0.29 | 173.60 | 0.23 | 136.95 | Lognormal |
| 35-44 years | 0.25 | 153.96 | 0.27 | 164.65 | Lognormal |
| 45-54 years | 0.28 | 171.48 | 0.33 | 197.45 | Lognormal |
| 55-64 years | 0.26 | 159.23 | 0.31 | 189.83 | Lognormal |
| 65-74 years | 0.31 | 185.20 | 0.26 | 159.15 | Lognormal |
| 75-100 years | 0.41 | 246.19 | 0.33 | 202.03 | Lognormal |
| Average in the LiveLighter® survey population | 0.28 |  | 0.28 |  |  |
| Notes: kJ: kilojoule; * Lognormal distribution defined by mean and standard errors from Australian Bureau of Statistics (ABS) mean sweet food consumption data.  Source: ABS 2013 [2] | | | | | |

**References**

1. Australian Bureau of Statistics. Microdata: National Health Survey, 2017-18. In: Australian Bureau of Statistics, editor. Canberra: DataLab; 2019.

2. Australian Bureau of Statistics. Microdata: Australian Health Survey, National Health Survey, 2011-12. In: Australian Bureau of Statistics, editor. Canberra: DataLab; 2013.
